# How does ‘banter’ influence trainee doctors’ choice of career? A qualitative study

**DOI:** 10.1186/s12909-019-1531-0

**Published:** 2019-04-11

**Authors:** David Wainwright, Michael Harris, Elaine Wainwright

**Affiliations:** 1 Department for Health, University of Bath, Claverton Campus, Bath, BA2 7AY UK; 2 Department of Psychology, Bath Spa University, Bath, UK; 3 Institute of Primary Health Care Bern (BIHAM), University of Bern, Bern, Switzerland

**Keywords:** Career choice, Medical education, Role model, Mentoring, Under recruitment, Job satisfaction

## Abstract

**Background:**

Negative comments from senior colleagues about specialties, such as general practice and psychiatry, are known to influence trainees’ career choice, but little is known about the extent of this influence or the mechanism by which it works. There have been calls to ban these disparaging comments, also known as ‘banter’. This study explored how recently qualified doctors make sense of banter in the context of other experiences and information.

**Methods:**

Semi-structured telephone interviews were conducted with 24 trainee doctors in their second postgraduate year in South West England. Thematic Analysis was used to code the data and organise them into themes.

**Results:**

Trainees are commonly exposed to banter about the merits of different specialties and those who work in them, but these messages are not received uncritically and are not perceived to be decisive in determining career choice. The views of senior doctors are assimilated with other experiences and information, as trainees strive to assess their ‘fit’ with a specialty. While banter is seen as positioning specialties in a status hierarchy, other factors such as work-life balance and feeling ‘at home’ in a specialty are often believed to be more significant factors in career choice. We posited two theories of banter; the ‘propaganda model’ and the ‘person-specialty fit model,’ and found the latter to provide a better understanding of how banter informs career choice.

**Conclusions:**

Banter often comprises stereotypes and caricatures, but despite its biases and distortions, it may still aid career choice. The challenge is not to ban banter, but to provide more accurate and reliable knowledge and experiences of what working life is like in different specialties.

**Electronic supplementary material:**

The online version of this article (10.1186/s12909-019-1531-0) contains supplementary material, which is available to authorized users.

## Background

Many factors can shape a junior doctor’s choice of career. Some fix on a particular specialty before starting medical school, [1] while the undecided must glean what they can from the curriculum content, their experience of placements, special studies modules, and the influence of faculty members, before reaching their decision. [1–3] These experiences can only provide a limited and partial understanding of working life in a particular specialty, and an individual’s suitability for it. Many trainees change their minds, with one study finding that 37% of doctors had altered their choice of specialist training during their first two postgraduate years. [4] In another study, over a quarter of recently graduated doctors who had seriously considered a specialty did not subsequently pursue that career. [5]

Faced with a major decision under conditions of uncertainty, the influence of peers and older more experienced doctors may prove decisive. A ‘hidden curriculum’ in which the values transmitted to students undermine the formal messages of the declared syllabus has been described. [6] One vector of transmission for the hidden curriculum might be ‘banter’, which comprises informal exchanges about the characteristics of different specialties and those who practise within them. [7]

Comments about intended careers can be both positive and negative [8], but attention has focused on how medical students’ career choices are affected by hearing disparaging and negative comments about some specialities, with 17–24% of students reporting that ‘badmouthing’ had influenced their career choices. [7, 9] While this occurs across all specialities [9, 10], it is reportedly a particular problem for general practice [7, 10, 11] and psychiatry [7, 10, 12, 13].

A study of UK medical students identified the low status of general practice, linked to a prevailing medical school culture, as one of the factors putting them off considering general practice as a career. [14] In a recent qualitative study of doctors’ career plans in their first UK postgraduate year, known as Foundation Year 1, the lack of respect for GPs from other doctors was one of the themes that emerged, and a ‘stigma’ of being a GP experienced in medical schools and hospitals was seen as a deterrent to a career in general practice [14]. This has resulted in calls for a ‘zero-tolerance’ policy regarding negative comments about general practice as a career [8, 15].

We know relatively little about the mechanism by which banter influences the career choice of young doctors. Most empirical studies assume that disparaging comments, about specialties and their practitioners, perpetuate a status hierarchy, with high-technology medicine and surgery at the top and general practice and psychiatry at the bottom, which skews trainees towards what they perceive to be high status specialties. [7, 13] Although not referred to as such, this is essentially a propaganda model. [16]

The ‘propaganda’ model of banter assumes that the primary motive of trainees is the desire for status and prestige, and that they unquestioningly accept the claims made in banter. Yet we know little about trainees’ desire to position themselves in such hierarchies, or, how they interpret and make sense of the claims made through banter in the context of other experiences and sources of information. Research in organisational psychology and human resource management suggests that a far wider range of factors shapes career choice, many of which are encompassed within the theory of ‘person-environment fit’, [17].

In a recent meta-analysis, person-environment fit was *“broadly defined as the compatibility between an individual and a work environment that occurs when their characteristics are well matched”* [18:281]. The work environment can be broken down into different domains - job, organisation, group, supervisor – within which the individual may fit to a greater or lesser degree. While recruiting employees who fit the work environment is important to employers, potential employees must also make this assessment in order to find a satisfactory career path, particularly where specialist training is required.

Recently qualified doctors, choosing which specialty to train for, have several sources of information to call upon to make this assessment, for example, we know that for those considering general practice the experience [19] and quality [15] of primary care placements, the duration of those placements [20], general practitioner role models [8], longitudinal GP placements [20], and the medical school’s primary care culture [19] are of particular importance. [15, 19–22] These factors have an objective reality, but how the trainee makes sense of them and invests them with meaning is a subjective process influenced by the interpretations of others. [23]

How young doctors use the information gleaned from banter, in the context of other experiences and sources of knowledge, to assess their fit within a given specialty, is an important topic, because the consequences of what might be termed poor person-specialty fit can be problematic for the individual and the organization. In a meta-analysis, [18] strong correlations between person-environment fit and a range of pre-entry and post-entry consequences were reported. Extrapolating from this meta-analysis suggests that doctors who enter a specialty that is a poor fit for them are likely to experience: low job satisfaction, job strain, failure to progress, and other negative outcomes that might lead them to leave the profession. While for the organization, poor person-specialty fit could be a cause of poor performance, which affects the quality of care, and may also lead to under-recruitment to some specialties.

While the evidence suggests that banter can influence medical career choices, little is known about the process by which this influence is exercised: what ‘banter’ comprises, to what extent it serves to position specialties in a hierarchy, how trainees make sense of it, whether they take it at face value or question its claims, and how influential it is perceived to be relative to other factors that shape their career choices.

In order to address these gaps in the evidence, we conducted a study of doctors in their second year of the Foundation Programme (FY2), the point at which most UK trainees make their final career choice, investigating the extent to which they had experienced ‘banter’ about their intended specialities, and whether/how they believed that this had affected their choice of career.

## Methods

### Research design

Semi-structured qualitative telephone interviews. We used an open-ended qualitative approach to allow more in-depth exploration of participants’ personal experiences and decision-making than would be possible using more structured methods.

### Participants and recruitment

The sampling frame (*n* = 262) comprised doctors who had completed their medical under-graduate degree and were in their FY2 post in the Severn region of the UK, at the time of recruitment. Administrators emailed all FY2 doctors, providing a study information pack and the research team’s email address. Potential participants were invited to contact the team for further information, before being asked to give informed consent. All those who contacted the researchers participated. A gift voucher was offered in token of thanks for participation.

We interviewed until the team agreed that no new themes were arising, (*n* = 24), [24].

### Data collection

The interview schedule was informed by our literature review (see Additional file 1). No substantial changes were needed after piloting with two FY2 doctors. The semi-structured interview schedule enabled us to respond to participants’ leads while gaining similar information from all participants. Interviews were conducted at times that suited the participants and lasted on average 40 min. All interviews were digitally recorded and transcribed verbatim. Transcripts were anonymised before data analysis.

### Data analysis

Thematic analysis was used to structure data collection and interpretation. [25, 26] The analysis proceeded sequentially, so that early findings could inform later interviews by modifying the schedule of questions. An inductive approach was adopted, so that the themes identified emerged from the data rather than from a pre-conceived coding scheme. Before coding, the researchers immersed themselves in the data through repeated readings of the interview transcripts, and initial interpretations were noted and discussed. All transcripts were coded by at least two members of the research team. The research team compared their interpretations, and jointly reviewed and refined themes and sub-themes, debating differences until consensus was reached and they had defined, named and exemplified all the themes. Quotations have been selected to illustrate key themes, and the number attached to each quotation is a unique participant code.

## Results

Twenty of 24 participants (83%) were female; nationally, 57.4% of FY2 doctors were female in 2014/5. [27]

### The nature of banter and the hierarchy of specialties

‘Banter’ about different specialties was often presented in a humorous form and comprised stereotypical accounts of those specialities. Such comments were often a vehicle for professional rivalry, particularly from and about surgeons:

I don’t think they were serious. It is always playing into a culture, like surgeons are cantankerous and egotists - that is the caricature of a surgeon in kind of doctor land. And I think the caricature of psychiatrists is they are like loopy, not real medics and kind of like soft doctors. (#6).

The rivalry between surgeons and hospital medical specialists has a long history reflecting their different professional origins [28]. Both parties had equally disparaging caricatures of the other, and these were used to suggest that a particular student or trainee was ‘too good’ for other specialties, in the following example, too good for surgery:

I remember [..] consultants asking me what I wanted to do, and me saying I wasn’t completely sure but I was thinking about surgery, and them saying “oh my god no, you’d be wasted as a surgeon, you have to do medicine. You’d just be completely wasted.” (#15).

The ‘too good for’ meme was applied to several specialties, but with particular force towards general practice and psychiatry, with some suggesting that the latter was not really medicine at all:

I suppose most of what people who are not psychiatrists have said would put people off as it is a much maligned, well fairly maligned specialty. If you say you want to do psychiatry to your educational supervisor you get a funny look and they [say], ‘Oh why? You are too good to be a psychiatrist.’ Or, ‘It is not real medicine.’ (#1).

Lack of expertise figured prominently in reports of banter about GPs, partly stemming from the specialist’s contempt for the generalist, who is seen as having wide-ranging, and hence relatively superficial knowledge*.* The following quotation notes the shorter duration of GP specialist training compared to other specialties, and links it to claims of inappropriate referrals, the latter being reported by several participants as a common gripe of hospital doctors against their GP colleagues:

[O]ne of your more senior doctors might say, “Why has the GP referred? This is such a bad referral letter. Why?” Especially, I think when I was on surgery as well, I remember one consultant said that he’d got to where he was after 10, 15 years of training, whereas a GP only had 3 years of training post-foundation, implying that they were much more inexperienced and less medically able. (#3).

Beneath the veneer of comic stereotypes and caricatures there often lies a more serious intention to position different specialties in a status or competence hierarchy. Hospital-based specialties, which require long periods of training and which are characterised by high technology medicine, specialist physiological knowledge or surgical techniques, were perceived to be close to the top of this hierarchy, while others, most notably general practice and psychiatry, were at the bottom. When we probed participants’ accounts of hierarchy more deeply, however, a more nuanced picture emerged, comprising multiple hierarchies based on very different criteria. Thus, specialties were not just ordered in a competence hierarchy, but also in terms of job demands, competition for places, and work-life balance, which tend to be an inversion of the competence hierarchy:

Psychiatry is perhaps dismissed by quite a lot of people and in my perception seems to be fairly low down at the bottom of the hierarchy if there is a hierarchy. But I suppose the hierarchy as such isn’t a straight unitary thing. There are different things like I suppose how difficult specialties are perceived to be and there is also how nice it would be to work in. … So I know a lot of people respect emergency medics because they are cool people and they obviously work incredibly hard and it is a very impressive thing to do and be a part of. But most people don’t want to do it as they know how hard it is for the work/life balance. (#1).

The opportunity to conduct more lucrative private work was mentioned by three trainees, (#11, #22, #24) but generally, variations in financial rewards were not discussed, although participants may have considered this to be taboo.

Many participants were uncomfortable with the concept of a hierarchy of specialties. Some denied there was a hierarchy, others challenged the validity of the perceived hierarchy, but then went on to position different specialties in hierarches of status, competence, job demands and work-life balance. The sense was not of one fixed or objective hierarchy, but of multiple hierarchies, dependent upon multiple factors..

I thought that the training pathways for different specialties is so different that - In some ways I feel that it’s non comparable, […] so I feel like everyone is very good at their own thing and that’s - I suppose that’s the point of the training pathways ... I don’t necessary feel like there is a hierarchy in my mind at all. (#14).

### Perceived influence on choice of specialist training

When asked directly, some respondents were trenchant in their claim that banter had not influenced their career choice:

It wouldn’t matter to me about a reputation or what people think of a certain specialty. If something interested me, it wouldn’t bother me what other people thought about it, I’d just do it. So that certainly doesn’t change my opinion of the specialty. (#3).

No participants reported that negative banter had been the primary factor in determining their choice of specialist training, but some suggested that it might have influenced their choice as an unconscious or background factor.

I think doing psychiatry there is definitely still a stigma to choosing it. And a lot of judgement on people who choose psychiatry. And I would like to say that doesn’t influence my decision, but I think in a way it does. How your peers see you and how they see the value of your career choices and work is important. (#9).

While the possibility of negative banter having a subliminal effect on specialty choice cannot be ruled out, what emerges from our data is that trainee doctors are not simply the passive recipients of banter, responding to its exhortations unquestioningly, but rather that they actively engage with the accounts of different specialties that are presented to them. Trainees were able to assess the veracity of the caricatures and stereotypes by comparing them with their own lived experiences:

I had decided I wanted to do psychiatry, and was very happy with that choice in second and third year pre-clinical, and most of those opinions [negative comments] were from other doctors in clinical years. So actually, I had already made up my mind I wanted to do that and it didn’t put me off because I had already found out a lot of those things are not really true. (#1).

For some participants, negative banter was perceived simply as falsehood, easily refuted by lived experience, but others had a more nuanced approach, suggesting that banter about different specialties may contain some truth, albeit in a distorted form:

… specialties are known for their character traits. In general, medics are people who like to take a problem and think about a problem and work out the answer to a problem, whereas surgeons are people who like to jump in and do things, and fix the problem. But I think that’s what people talk about, but I also think that’s true, that’s a realistic element of specialty that you have to consider. So I guess, yes I’ve spoken to people about specialties, tried to work out what would suit me and my personality, but I don’t think the stereotypes and the banter and the hierarchy and stuff, I don’t think that’s really affected me. (#18).

### Person-specialty fit

Trainees asked themselves ‘Am I the right person for that job?’ in terms of several dimensions upon which they assessed their aptitudes, attitudes, beliefs and preferences. Adding further complexity, some trainees did not simply seek to answer these questions at a fixed point in time, but projected forwards to consider how their person-specialty fit might change across their career as their lives and circumstances changed. For some, competence and interest in the work were key criteria, but there was recognition that work-life balance might become more important in the future:

It’s something I’ve thought about a lot, and I wonder if I’ll regret that in 10 years’ time when I’ve got children. But I think at the moment, I have to follow what I feel I’ll be good at, and what I feel I’m interested in, and I think to do something I wasn’t, just so that I could have more time at home, I think I’d end up resenting that. (#18).

In order to answer these questions about person-specialty fit, trainees drew on two key sources of information: comments, advice, and guidance from others; and direct experience of a specialty, gathered during placements. Both sources could provide insights, but they could also be the source of bias, erroneous stereotypes and misinformation. Trainees were not so much looking for ‘hard facts’ about a specialty, but a more general sense of ‘fit’. Surgery is a good example of this process, as some participants reported being influenced by exposure to stereotypical assumptions about what makes a good surgeon:

I wasn’t particularly confident and if you said you wanted to be a surgeon they said, ‘Oh but you don’t look like a surgeon, you don’t act like a surgeon.’ So I almost became intimidated out of doing surgery. (#10).

Gender expectations and a lack of female surgeons as role models were key issues in our sample, but there was a broader issue about identification with the stereotype. Failure to identify could lead trainees to assume that a specialty was not for them.

I didn’t really feel like I was like the consultants, or anything, because there weren’t any women consultants there. So I felt I could see myself there, but there wasn’t anyone that I could be like, ‘I want to be like you,’ if that makes sense? (#12).

Strong identification with those already working in a specialty could prove decisive in career choice, not least because it made the trainee feel ‘at home’:

But I really liked the people I work with and they are exactly how I, yes they were exactly the kind of people that made me want to work in psychiatry in the first place. Really open minded and supportive and self-aware so that kind of confirmed it. (#6).

On placement, trainees did not simply learn about the job, they also learnt about themselves, their particular skills and aptitudes, their ability to relate to patients, and what they found enjoyable and satisfying:

I think it suits my natural abilities. And I feel that although I found the placement really hard and it wasn’t the job that I [initially] wanted to do, when I was in a room with a patient dealing with their distress and talking to them about what was happening in their lives, that felt like home to me. That felt like the place where I felt competent and engaged and interested and yes so that felt like the right place to be. (#6).

For others, experience on placements could be off-putting, sometimes because their expectations were disappointed. For example, one participant (#5) found general practice to be a ‘lonely’ experience compared to hospital medicine, others found particular specialties ‘boring’, but also because the experience positioned them at a lower level in hierarchies of competence and commitment:

I remember, in surgery I did notice that those surgeons, what they did, they were fantastic, but I could never see myself doing that job. I think you need a certain amount of confidence in your own abilities, (which I never thought I had), to do surgery. And so I guess more so it was the opposite, there were things I thought I could definitely not do this and that was mainly in those surgical specialities. (#8).

General practice occupied an unusual position in terms of the search for person-specialty fit. While there were aspects of the job that participants liked or did not like, the dilemma over person-specialty fit seemed different to that for most other specialties. For some it was seen as the ‘default’ or ‘fall-back’ career choice – what one chose in the absence of a strong identification with another specialty, or if entry to more competitive specialist training programmes were unsuccessful. For some, the perception that general practice was what people settle for if other plans go awry could in itself be off putting:

I hate to say it, but I think with GP a lot of people say you fall into it if you don’t know what to do, that’s why you end up in GP. That’s had a bit of a negative impact for me because I think, “Well, I don’t want to end up in a specialty.” I want to work really, really hard and challenge myself and achieve a goal. And I don’t necessarily feel like I personally would get that from GP. […] And a lot of my friends have also felt, “Oh, I don’t know what to do. I’ll probably just become a GP.” […] if you don’t know what to do it’s almost the default kind of job. (#2).

## Discussion

### The content of banter

Our findings suggest that banter about the merits and demerits of different specialties and the people who work in them is commonly encountered by trainee doctors. The content of banter is diverse but the more significant themes concern variations between specialties relating to: intellectual demands, workload, work/life balance, and the personality attributes they require. These representations of different specialties can also be linked directly to individual trainees, in statements about what a particular doctor is *‘too good for’,* or for which she or he would be well-suited.

### The propaganda model

We have found some evidence for the propaganda model of banter. However, while some senior doctors do make negative comments about some branches of medicine and their practitioners, often placing general practice and psychiatry at the bottom of a putative status hierarchy, trainees are not simply the passive recipients of these messages, unquestioningly accepting their veracity. Instead, young doctors appraise the information presented to them in banter alongside that from other sources and experiences. Rather than accepting the putative status hierarchy and aiming for the most prestigious jobs, trainees consider a range of factors relating to the characteristics of different specialties and their suitability for them.

### The influence of banter on career choice and person-specialty fit

Banter is often seen as light-hearted, and a key finding is that many participants claimed that its influence on their career choice was, at most, marginal. Negative banter does serve to position different specialties in terms of broadly accepted notions of status or prestige, but this position does not translate directly into the perceived desirability of working in a particular specialty; it is possible to have a ‘bad’ job in a high status specialty, or a ‘good’ job in a low status specialty. Moreover, trainees do not simply accept banter as a true representation, but compare what they hear about a specialty with the experiences they have on placements, often leading them to challenge the accuracy of caricatures, or question the values and assumptions that underpin notions of hierarchy, for example, by elevating the complexity and expertise of general practice or psychiatry over that of allegedly more prestigious specialties.

Although banter can be inaccurate or biased, it is used by trainees as a source of tacit knowledge about everyday working life within a particular specialty, and their personal suitability to work within it. Along with other sources of information and personal experiences gleaned from placements, banter is used by trainees to assess their person-specialty fit. Given the importance to trainees of assessing person-specialty fit before choosing specialist training, it is unlikely that banter can be eradicated, and a more promising strategy might be to counter the biases of banter with more objective and balanced careers guidance.

### Linking with previous studies

Other studies have suggested that negative banter, or ‘bashing’ of specialties is common, with a recent survey finding that 67% of medical students reported personally receiving ‘non-constructive criticism about their preferred specialty’, while 79% ‘believed bashing was unprofessional behaviour’ [10]. Our findings also suggest that negative banter and claims of a hierarchy of specialties, are not viewed positively by trainees, but the trainees’ response to banter is rather more nuanced than this critical stance implies. Banter can represent a distorted picture of the challenges and demands of different specialties and the qualities and attributes of the doctors who work in them, but while banter may deal in caricatures, trainees perceive that it is not entirely divorced from reality. For example, in some specialties the work-life balance really is weighted more heavily on one side than the other, and certain personal attributes and characteristics really might make a trainee more suitable for one or another speciality. By articulating an image of person-specialty fit, albeit distorted, banter provides one of the resources by which trainees can obtain tacit knowledge of whether a specialty is right for them. A recent survey of physicians and medical students [7] also found an equivocal, or “confused” perspective on banter: while 81% condemned it as unprofessional, 72% saw it as ‘an integral part of medicine’, with some suggesting that it only affects ‘those who are insecure about choice of specialty.’

If banter provides the medium through which older and more experienced doctors share with trainees their thoughts on person-specialty fit, then the challenge is not to ban banter but to complement it with more accurate information about the experience of working in different specialties and the qualities required to achieve a satisfying and rewarding career within them. For example, other studies have reported that for general practice, the presence [21] and quality [15] of students’ primary care placements, the duration of those placements [20], general practitioner role models [8], longitudinal GP placements [20], and the medical school’s primary care culture [19] are of particular importance.

### Implications for practice

Our findings suggest some points of intervention where the received wisdom expressed in banter might profitably be contested. The first is the gendered stereotype of the surgeon; while self-confidence and decisiveness may well be essential attributes for the specialty, they should not be seen as exclusively male. Secondly, the perception that psychiatry lacks complexity or lies outside of ‘real medicine’ could be countered with more accurate information about the efficacy of psychiatric interventions and the difference they can make to patients’ lives. Thirdly, the perception of general practice as a ‘default’ job or ‘fall-back’ career may be expressions of a ‘hospital-centric culture,’ [29], which may require a more radical reform of medical education to overcome. Further research is needed to explore how the distortions and biases expressed in banter can be challenged by a richer and more accurate appraisal of person-specialty fit achieved through medical education and placements.

### Strengths and limitations of the study

The use of qualitative interviews enabled us to explore participants’ experiences of banter in greater depth than a quantitative study would have allowed. This enabled us to explore some of the ambiguities and contradictions of banter, revealing how our participants made sense of banter in the context of other experiences, and used these multiple sources of information to guide their career choices.

While data saturation was achieved, our sample may not have been representative of all FY2 trainees. Women were significantly over-represented, and it might be that exposure to banter and the influence it has on career choice is different for male trainees. We do not know why we attained a gender imbalance in response to our study invitation. Researchers have found that offering incentives means that women in the general population complete more telephone survey items than men [30] but we found no evidence that female doctors are more likely to respond to a research interview request than their male colleagues. The study was conducted in a single Deanery in South-West England, and this limits the transferability of the findings. In asking participants whether banter influenced choice of specialist training, there is the possibility of social desirability bias in the responses, particularly in a profession that values self-efficacy and independence of mind: respondents might have been reluctant to admit that their choices were influenced by banter, thus our findings may understate the influence of banter on career choice.

## Conclusion

Previous research has suggested that junior doctors’ career choices are influenced by negative comments about some specialties in the form of ‘banter’ from older colleagues. We have explored how trainees use this information to inform their decision-making. Where others have adopted a propaganda model, our findings reveal a more active and critical engagement, in which claims made in banter are tested against experiences from placements and other sources of information, which is more consistent with a person-specialty fit model. In choosing which specialty to pursue, trainee doctors must answer two important questions: What will working in this specialty be like? And, am I the right sort of person for the job? Banter provides a source of information, albeit sometimes distorted or biased, on which answers to these questions can be based. The influence of banter on career choice may be more limited and more nuanced than previous studies have implied.

## Additional file

Additional file 1: Trainee_dr_career_choice_interview_schedule_v1.docx This file contains the interview schedule used in the study. (DOCX 21 kb)

## References

[1] Johnson AL, Sharma J, Chinchill VM, Emery SE, McCollister Evarts C, Floyd MW (2012). Why do medical students choose orthopaedics as a career?. J Bone Joint Surg.

[2] Farooq K, Lydall GJ, Malik A, Ndetei DM, Bhugra D (2014). Why medical students choose psychiatry - a 20 country cross-sectional survey. BMC Med Educ.

[3] Avery D, Wheat J, McKnight J, Leeper J (2009). Factors associated with choosing family medicine as a career specialty: what can we use?. Am J Clin Med.

[4] UK Foundation Programme Office. Career destination report 2016. Foundation Programme Office 2016 http://www.foundationprogramme.nhs.uk/sites/default/files/2018-08/2016%20F2%20Career%20Destinations%20Report.pdf Accessed 26 Oct 2018.

[5] Goldacre MJ, Goldacre R, Lambert TW (2012). Doctors who considered but did not pursue specific clinical specialties as careers: questionnaire surveys. J Royal Soc Med.

[6] Mahood SC (2011). Medical education: beware the hidden curriculum. Can Fam Physician.

[7] Ajaz A, David R, Brown D, Smuk M, Korszun A (2016). BASH: badmouthing, attitudes and stigmatisation in healthcare as experienced by medical students. BJPsych Bull.

[8] Alberti Hugh, Banner Kimberley, Collingwood Helen, Merritt Kymberlee (2017). ‘Just a GP’: a mixed method study of undermining of general practice as a career choice in the UK. BMJ Open.

[9] Hunt DD, Scott C, Zhong S, Goldstein E (1996). Frequency and effect of negative comments ("badmouthing") on medical students' career choices. Acad Med.

[10] Holmes D, Tumiel-Berhalter LM, Zayas LE, Watkins R (2008). "Bashing" of medical specialties: students' experiences and recommendations. Fam Med.

[11] Bellodi PL (2004). The general practitioner and the surgeon: stereotypes and medical specialties. Rev Hosp Clin Fac Med Sao Paulo.

[12] Gaebel W, Zaske H, Zielasek J, Cleveland HR, Samjeske K, Stuart H (2015). Stigmatization of psychiatrists and general practitioners: results of an international survey. Eur Arch Psychiatry Clin Neurosci.

[13] Baker M, Wessely S, Openshaw D (2016). Not such friendly banter? GPs and psychiatrists against the systematic denigration of their specialties. Br J Gen Pract.

[14] Merrett A, Jones D, Sein K, Green T, Macleod U (2017). Attitudes of newly qualified doctors towards a career in general practice: a qualitative focus group study. Br J Gen Pract.

[15] Nicholson S, Hastings AM, McKinley RK (2016). Influences on students' career decisions concerning general practice: a focus group study. Br J Gen Pract.

[16] Ellul J. Propaganda: The Formation of Men's Attitudes. Trans. Kellen TK, Lerner J, The formation of Men's attitudes. New York: Vintage Books; 1973.

[17] Schneider B (2001). Fits about fit. Appl Psychol-Int Rev.

[18] Kristof-Brown AL, Zimmerman RD, Johnson EC (2005). Consequences of individuals’ fit at work: a meta-analysis of person-job, person-organisation, person-group, and person-supervisor fit. Pers Psychol.

[19] Erikson CE, Danish S, Jones KC, Sandberg SF, Carle AC (2013). The role of medical school culture in primary care career choice. Acad Med.

[20] Amin M, Chande S, Park S, Rosenthal J, Jones M (2018). Do primary care placements influence career choice: what is the evidence?. Educ Prim Care..

[21] Wiener-Ogilvie S, Begg D, Dixon G (2015). Foundation doctors’ career choice and factors influencing career choice. Educ Prim Care.

[22] Barber S, Brettell R, Perera-Salazar R, Greenhalgh T, Harrington R (2018). UK medical students’ attitudes towards their future careers and general practice: a cross-sectional survey and qualitative analysis of an Oxford cohort. BMC Med Educ.

[23] Middleton D, Engeström Y (1998). Cognition and communication at work.

[24] Baker SE, Edwards R, Doidge M. How many qualitative interviews is enough? Expert voices and early career reflections on sampling and cases in qualitative research. National Centre for Research Methods Review Paper. 2012. http://eprints.ncrm.ac.uk/2273/4/how_many_interviews.pdf Accessed 26 Oct 2018.

[25] Aronson J (1995). A pragmatic view of thematic analysis. Qual Rep.

[26] Braun V, Clarke V (2006). Using thematic analysis in psychology. Qual Res Psychol.

[27] The UK Foundation Programme Office. FP annual report 2015. 2015. http://www.foundationprogramme.nhs.uk/sites/default/files/2018-09/2015%20FP%20Annual%20Report.pdf. Accessed 26 Oct 2018.

[28] Porter R (1997). The greatest benefit to mankind: a medical history of humanity from antiquity to the present.

[29] Petchey R, Williams J, Baker M (1997). Ending up a GP': a qualitative study of junior doctors' perceptions of general practice as a career. Fam Pract.

[30] Singer E, van Hoewyk J, Maher MP (2000). Experiments with incentives in telephone surveys. Public Opin Q.

