# Correction to: How does ‘banter’ influence trainee doctors’ choice of career? A qualitative study

**DOI:** 10.1186/s12909-019-1707-7

**Published:** 2019-08-20

**Authors:** David Wainwright, Michael Harris, Elaine Wainwright

**Affiliations:** 1 Department for Health, University of Bath, Claverton Campus, Bath, BA2 7AY UK; 2 Department of Psychology, Bath Spa University, Bath, UK; 3 Institute of Primary Health Care Bern (BIHAM), University of Bern, Bern, Switzerland

**Correction to: BMC Med Educ**

**https://doi.org/10.1186/s12909-019-1531-0**

Following publication of the original article [1], the authors reported an error in the first paragraph of the ‘Results’ section.

**First paragraph of ‘Results’ section in the original article:**

Twenty of 24 participants (83%) were female; nationally, 57.4% of FY2 doctors were female in 2014/5.

**Corrected first paragraph of ‘Results’ section:**

Seventeen of 24 participants (71%) were female; nationally, 57.4% of FY2 doctors were female in 2014/5.

## References

[1] Wainwright (2019). How does ‘banter’ influence trainee doctors’ choice of career? A qualitative study. BMC Med Educ.

