# Supplementary material for: How does ‘banter’ influence trainee doctors’ choice of career? A qualitative study
Source: BMC Med Educ. 2019 Apr 11;19:104. doi: 10.1186/s12909-019-1531-0 (PMC6460642; doi:10.1186/s12909-019-1531-0)
Supplement: Supplementary file 1 — Trainee_dr_career_choice_interview_schedule_v1.docx This file contains the interview schedule used in the study. (DOCX 21 kb) [file 12909_2019_1531_MOESM1_ESM.docx]

**Trainee doctors’ career choice study: interview schedule**

1. Tell me a bit about your medical career so far – the medical school that you went to, the jobs you’ve done so far etc.
2. Thinking back to when you were a medical student, or even before that – did you have any thoughts then as to what specialty you would eventually like to go into? What do you think influenced that?
3. How have your ideas on the specialty you’d like to go into changed over time? What has caused that change?
4. Have you already made a final decision about the speciality that you’d like to enter? When did you make that decision, and what triggered it? Are there any other specialties that you seriously considered? What made you decide not to go for those?
5. How much have other people’s ideas and comments affected the way you think, or thought, about the different specialties? Can you give me some examples?
6. It has been suggested that there is a hierarchy of specialties, have you encountered that viewpoint? [Prompts: who from: patients, trainees, senior health professionals].
7. Do you yourself feel that some specialties deserve to be more prestigious than others? [Prompts: Why? Demands, pressures, complexity?]
8. [Only ask if answered yes to 6 and/or 7] Can you give me some examples of the experiences that made you aware of this hierarchy of specialties? [Prompts: comments by patients/trainees/others; experiences on rotations; experience of learning their knowledge base].
9. [Only ask if answered yes to 6 and/or 7] Which specialties are at the top of the medical hierarchy and which are at the bottom? [Prompts: where would you place general practice?]
10. [Only ask if answered yes to 6 and/or 7] Has the hierarchy of specialties influenced your preferences regarding specialist training and career path? [Prompts: How?].
11. Have you ever heard particularly positive, or negative, comments from your more senior colleagues about other specialties? How did people react to those? How much have they affected your own thinking?
